# A Dual Protective Effect of Intestinal Remote Ischemic Conditioning in a Rat Model of Total Hepatic Ischemia

**DOI:** 10.3390/jcm8101546

**Published:** 2019-09-26

**Authors:** Zoltan Czigany, Koichiro Hata, Wei Lai, Timo Schwandt, Yuzo Yamamoto, Shinji Uemoto, Rene H. Tolba

**Affiliations:** 1 Department of Surgery and Transplantation, University Hospital RWTH Aachen, 52074 Aachen, Germany; 2 Department of Surgery, Division of Hepato-Biliary-Pancreatic Surgery and Transplantation, Kyoto University Graduate School of Medicine, Kyoto 606-8507, Japan; khata@kuhp.kyoto-u.ac.jp (K.H.); uemoto@kuhp.kyoto-u.ac.jp (S.U.); 3 Organ Transplantation Department, Tongji Hospital, Wuhan 430030, China; weilai@tjh.tjmu.edu.cn; 4 Institutes of Molecular Medicine and Experimental Immunology (IMMEI), University of Bonn, 53127 Bonn, Germany; timo.schwandt@roche.com; 5 Department of Gastroenterological Surgery, Akita University Graduate School of Medicine, Akita 010-8543, Japan; yy@med.akita-u.ac.jp; 6 Institute for Laboratory Animal Science and Experimental Surgery, RWTH-Aachen University, 52074 Aachen, Germany

**Keywords:** liver ischemia, total hepatic ischemia, ischemia, reperfusion, remote conditioning, ischemic conditioning, intestinal conditioning

## Abstract

The present study aimed to investigate the effects of intestinal remote ischemic preconditioning (iRIC) on ischemia-reperfusion injury (IRI) and gut barrier integrity in a rat model of total hepatic ischemia (THI). Male Wistar rats (*n* = 50; 250–300 g) were randomly allocated into two experimental groups: RIC/Control. Thirty minutes of THI was induced by clamping the hepatoduodenal ligament. iRIC was applied as 4-min of ischemia followed by 11-min of reperfusion by clamping the superior mesenteric artery. Animals were sacrificed at 1, 2, 6, 24 h post-reperfusion (*n* = 5/group/timepoint). RIC of the gut significantly improved microcirculation of the ileum and the liver. Tissue ATP-levels were higher following iRIC (Liver: 1.34 ± 0.12 vs. 0.97 ± 0.20 μmol/g, *p* = 0.04) and hepatocellular injury was reduced significantly (ALT: 2409 ± 447 vs. 6613 ± 1117 IU/L, *p* = 0.003). Systemic- and portal venous IL-6 and TNF-alpha levels were markedly lower following iRIC, demonstrating a reduced inflammatory response. iRIC led to a structural and functional preservation of the intestinal barrier. These results suggest that iRIC might confer a potent protection against the detrimental effects of THI in rats via reducing IRI and systemic inflammatory responses and at the same time by mitigating the dramatic consequences of severe intestinal congestion and bacterial translocation.

## 1. Introduction

Ischemia-reperfusion injury (IRI) is inevitably encountered in various clinical scenarios in liver transplantation and oncological liver surgery, representing an important risk factor for inferior outcomes with increased morbidity and mortality, prolonged intensive care/in-hospital stay, and a significant increase of costs [1,2].

Following a landmark observation of Toledo-Perayra et al., demonstrating the presence of ischemic-reperfusion injury in transplanted livers of dogs in 1975, several methods have been introduced to reduce hepatic IRI in experimental and clinical settings [1,3,4,5,6,7].

Remote ischemic conditioning (RIC) was introduced by Przyklenk et al. in 1993, showing for the first time that brief ischemic-reperfusion attacks, applied at a remote organ or tissue (e.g., limbs or intestine), can protect certain target organs against the deleterious effects of IRI via triggering various protective pathways [8,9]. Although the RIC technique may be a powerful tool against the effects of IRI in different experimental models and clinical scenarios, the exact underlying mechanisms and the definitive explanation of the phenomenon still remain unclear [10]. Although, our group and others have intensively investigated the effects of RIC applied on the skeletal muscle in partial hepatic ischemia and liver transplantation [10,11,12,13,14,15,16], only very limited data is available on the effects of intestinal RIC (iRIC) in total hepatic ischemia (THI) [17,18]. Longer periods of THI without porto-systemic shunt, result not only in an expressed hepatocellular damage but also lead to a severe intestinal congestion and injury of the small bowel mucosa with consequential loss of barrier function and bacterial translocation. Therefore, prolonged periods of THI of over 30-min are considered to be lethal in rats, leading to high mortality rates without intervention [17,19,20].

Due to this above-described dual injury (liver IRI and splanchnic congestion) induced by THI, we hypothesized that iRIC might confer a protection via local conditioning effects on the small intestine, protecting against the dramatic consequences of the loss of barrier function and bacterial translocation as well as by mitigating hepatic IRI, targeting the liver as a remote organ.

This study was designed to investigate the effects of iRIC on hepatic and intestinal injury in a rat model of THI. Various parameters, known to be relevant in IRI and RIC, were used to assess intestinal and hepatic injury, systemic inflammation, and protective responses following THI and iRIC treatment.

## 2. Materials and Methods

### 2.1. Animals

All experiments were performed in accordance with institutional guidelines and the German federal law regarding the protection of animals. The ethical proposal of the study was approved by the responsible authorities (Bezirksregierung Köln, Cologne, Germany, ID: 50.203.2BN45). All animals received human care according to the principles of the “Guide for the Care and Use of Laboratory Animals” (8th Edition, NIH Publication, 2011, USA). The present study was designed, performed and reported according to the principles of the ARRIVE (Animal Research: Reporting of In Vivo Experiments) guidelines [21].

Male Wistar rats (RjHan:WI; Janvier Labs, Le Genest Saint Isle, France) were used (Σ*n* = 50; body weight range: 250–300 g). The animals were housed under specific pathogen-free conditions according to the guidelines of the “Federation for Laboratory Animal Science Associations” (FELASA; www.felasa.eu) with a 12-h light and dark cycle in a temperature- and humidity-controlled barrier environment. Water and standard pellets for laboratory rats (Sniff GmbH, Soest, Germany) were provided ad libitum.

### 2.2. Surgical Technique

To avoid disturbing effects of circadian rhythm, all experiments were performed at the same time of day, following an acclimatization period of one week. Volatile anesthesia was performed using 2 vol% isoflurane (Forane; Abbott GmbH, Wiesbaden, Germany) during all the surgical interventions. All surgical procedures were performed by the same surgeon.

**Figure 1:**
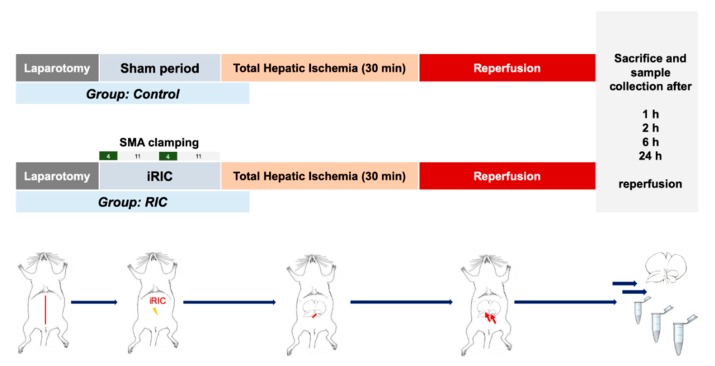
Study flowchart surgical protocol. Animals were randomized into two experimental groups (Control, RIC). Following laparotomy and dissection of the superior mesenteric artery (SMA) intestinal remote ischemic conditioning (iRIC) was applied as of 4-min of ischemia and 11-min of reperfusion via clamping of the SMA. Total hepatic ischemia was induced by clamping the hepatoduodenal ligament including both the portal vein and the hepatic artery. Animals were sacrificed after 1, 2, 6, 24 h of reperfusion for sample collection and further analysis (*n* = 5/group/time point). Modified from Emotzpohl, Czigany et al. Shock. 2018 [15]. Abbreviations used: iRIC—Intestinal remote ischemic conditioning; SMA—Superior mesenteric artery.

After sufficient anesthesia and analgesia (buprenorphine 0.03 mg/kg/24 h; Temgesic; EssexPharma, Haar, Germany), laparotomy was performed through a midline incision, the liver was mobilized by cutting its ligaments and the superior mesenteric artery (SMA) was exposed. Remote ischemic conditioning treatment was applied as 2 cycles of 4-min of ischemia and 11-min of reperfusion (total 30-min) by clamping of the SMA using an atraumatic microvascular clamp (Aesculap Yasargil FT260T; B.Braun) as described by our group previously on different occasions (Figure 1) [11,13,14,17]. Animals of the Control group underwent the exact same procedure without iRIC. Afterwards, THI was achieved by clamping the bilio-vascular pedicle of the whole liver using an atraumatic microvascular clip (FT260T) and ensuring that both the main portal vein and the hepatic artery are included. After 30-min of THI the clamp was removed to allow free reperfusion of the liver. No porto-systemic shunt was applied. At the end of the surgical procedure, the laparotomy (in 1, 2, 6, 24 h of reperfusion groups) was closed in two layers using 4-0 continuous sutures (Vicryl 4-0; Ethicon). 

Postoperatively, the animals were placed in an intensive care unit cage (Vetario; Brinsea Products Ltd., North Somerset, UK) for a recovery period of one hour, providing warmed air (30–35 °C) and an oxygen supply. After surgery, antibiotic treatment and analgesia were achieved by subcutaneous injections of cefuroxime sodium (16 mg/kg/24 h) (Cefuroxim Fresenius; Fresenius Kabi Deutschland GmbH, Bad Homburg, Germany) and buprenorphine (0.03 mg/kg/24 h). During the first 4-h postoperatively, animals were observed continuously and then transferred back to their cages and normal environment. Following the observation periods defined by the protocol, samples were collected, and animals were sacrificed subsequently under deep isoflurane anesthesia 2 vol%–4 vol% and buprenorphine (0.03 mg/kg) analgesia.

### 2.3. Experimental Design

For the present study 50 surgical procedures were performed based on an a priori sample size estimation. Recipients were randomly allocated into two experimental groups (*n* = 25 cases/group) (Figure 1).

Control: after dissection of the SMA and a corresponding sham waiting period of 30-min, no remote conditioning was applied and THI was induced as described above

RIC: remote ischemic conditioning protocol was applied as described above before THI.

After 1, 2, 6, and 24-h of portal reperfusion, liver and ileum microcirculation were measured in anesthesia (*n* = 5 cases/group/time point). Systemic- and portal venous blood from the vena cava and the portal vein as well as tissue samples from the liver (right mediate lobe) and from the ileum (2 cm proximal from the ileocecal valve) were collected for analysis before the animals were sacrificed via exsanguination in deep anesthesia. Five animals per group have been used for the in vivo imaging experiments. Figure 1. depicts a flowchart of the experimental protocol. 

During the survival period all animals were visited at least every 12-h by an experienced veterinary technician blinded for the experimental design and their clinical condition was evaluated using a human-endpoints score sheet. The score sheet was based on the previous work of Morton and Griffiths and the recommendations of our group for experimental studies in the field of liver research [21,22]. 

### 2.4. Liver and Ileum Perfusion

We evaluated hepatic and ileal microcirculatory perfusion using multiple timepoints at sacrifice, before collecting blood and tissue samples. As a reference control (baseline), we measured hepatic and ileal circulation in 10 rats just after laparotomy. The hepatic microcirculation (flow) and tissue oxygen saturation (StO_2_) were evaluated using an O2C device and a corresponding surface probe (O2C-oxygen to see device, LF1 surface probe; LEA Medizintechnik GmbH, Giessen, Germany) as described by our team previously [11,15]. The output signal was transferred to an integrated computer equipped with software to yield real-time display of data, and to record and analyze the blood flow pattern and values (LEA Medizintechnik GmbH, Giessen, Germany).

### 2.5. Biochemical Analysis and Serum Cytokines

Blood samples, collected from the inferior vena cava and from the portal vein by direct puncture with a 20-gauge needle at sacrifice, were centrifuged (room temperature, 10-min, 2500 rpm) and then serum levels of alanine aminotransferase (ALT), aspartate aminotransferase (AST), lactate dehydrogenase (LDH) were measured using an automated analyzer and standard photometric procedures (Vitros 250; Johnson and Johnson, Neuss, Germany). Serum samples, stored at −80 °C, were used for interleukin-6 (IL-6) and tumor necrosis factor alpha (TNF-α) assessments using commercial rat enzyme-linked immunosorbent assay (ELISA) kits (R and D Systems, Minneapolis, MN, USA) according to the manufacturer’s guidelines.

### 2.6. Tissue Adenosine Triphosphate Concentration

The apical part of the left lateral lobe was snap-frozen with liquid nitrogen pre-cooled metal tongs before sacrifice. Subsequently, the intestinal specimens were harvested from an identical anatomical location without mesenteric tissue (1 cm segment of the ileum 2 cm proximal from the ileocecal valve) and were immediately snap frozen in liquid nitrogen. Liver and intestinal tissues samples were stored at −80 °C until the assessment of adenosine triphosphate (ATP) concentrations, as described in detail elsewhere [11,23,24,25,26]. Briefly, the specimens were transferred into a vacuum freezer (Christ 2–16, Osterode, Germany) at −40 °C with a pressure less than 0.001 atm for at least 2 weeks of freeze-drying. After lyophilization, samples were homogenized and deproteinized and tissue ATP concentrations were determined by standard enzymatic tests [24,25,26]. The results were calculated and expressed as micromoles per gram of dry-weight.

### 2.7. Transmission Electron Microscopy

Following THI and 6-h of reperfusion, tissue samples of the ileum were immersed in a 2% glutaraldehyde and paraformaldehyde solution in phosphate-buffered saline. Following further sample preparation, as described before [27], specimens were examined using electron microscopy (EM400 T/ST, Philips, Amsterdam, The Netherlands).

### 2.8. Bioluminescent Assessment of Bacterial Translocation

Before surgery, animals (*n* = 5/group) have received a standard dose of bioluminescent *Escherichia coli* (*E. coli*), modified to contain the lux operon from P. luminescens, dissolved in phosphate-buffered saline (6 × 10^11^ in 0.5 mL i.g.; orogastral administration using feeding needles). The lux genes code for both the bacterial luciferase and substrate biosynthesis enzymes, which enable the strain to produce luciferase and its substrate simultaneously, thus no exogenous luciferin substrate was required [28].

Following surgery and 6 h of reperfusion the animals were re-anaesthetised and in vivo imaging was performed using the IVIS 100 System (Caliper Life Sciences Inc., Hopkinton, MA, USA) as described before [29,30,31]. Images were captured using the corresponding software provided by the manufacturer (Living Image Software 2.0, Caliper Life Sciences Inc.). The imaging system consists of a cooled charge-coupled-device camera mounted on a light-tight chamber, a camera controller, a cryogenic refrigeration unit connected to a computer system. Following in vivo imaging, animals have been sacrificed in deep anesthesia by removing the lungs, spleen, liver and mesenteric tissue including lymph nodes and the presence of bioluminescence has been directly analyzed to assess the translocation of labelled *E. coli* in distant organs following IRI.

### 2.9. Statistical Analysis

Results are expressed as mean ± standard error of the mean (s.e.m.) for each group. Two-way analysis of variance (ANOVA) and Bonferroni post-hoc test was performed to analyze changes in time dependent parameters and between group differences at each time point. Mann-Whitney-U test was applied to test the differences within two groups. Differences were considered significant when *p* < 0.05. Data plotting and analysis were performed using GraphPad Prism 8 (GraphPad Software Inc., San Diego, CA, USA) software package.

## 3. Results

### 3.1. Liver and Ileum Microcirculation

**Figure 2:**
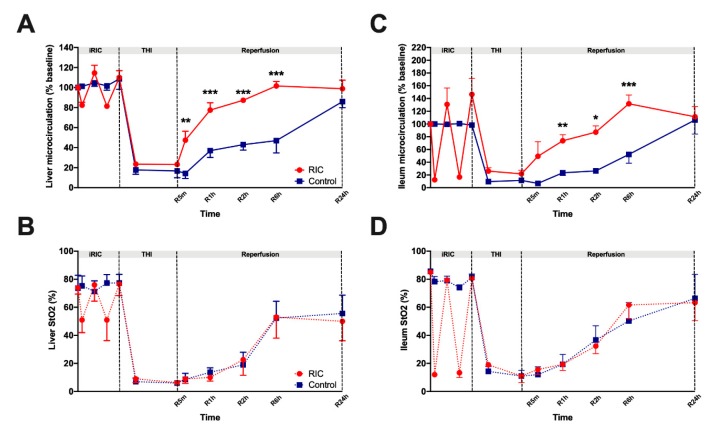
Microcirculation of the ileum and the liver. (**A**) Liver perfusion measured with the O2C device remained higher in the RIC group compared to Control throughout the reperfusion period. (**C**) Ileum perfusion remained higher in the RIC group compared to Control throughout the reperfusion period, however the significant difference between the two experimental groups has disappeared after 24-h of reperfusion. (**B**,**D**) Partially similar characteristic features were observed in terms of tissue oxygen saturation of the liver and ileum over the course of the observation period, however, StO_2_ values did not follow directly the positive alterations of the flow observed in the RIC group. Accordingly, no significant between group differences were detected. (mean ± s.e.m, * *p <* 0.05, ** *p* < 0.01, *** *p <* 0.001, RIC vs. Control, two-way ANOVA and Bonferroni post-hoc test, *n* = 5/group/time point). Baseline was determined as the mean flow measured in 10 healthy animals right after laparotomy. Abbreviations used: iRIC—Intestinal remote ischemic conditioning; THI—Total hepatic ischemia, StO_2_—Oxygen saturation.

The preischemic baseline hepatic and intestinal microcirculatory flow did not differ markedly between the two experimental groups (Figure 2). During the period of iRIC, flow values of all animals of the RIC group showed significant fluctuations which were more expressed in the ileal flow (flow 10–140%).

During the ischemic period, no significant differences were detected between the groups. After liver exclusion and the induction of THI, flow values of the liver and ileum have dropped dramatically. Relatively rapid recovery of the flow was observed after the reperfusion period in the RIC group. Over the early period of liver reperfusion flow values of both the liver and the ileum were significantly higher compared to the Control group (Figure 2). Meanwhile, the iRIC treated animals have reached flow-values comparable with the baseline values as early as after 2-h. Ileal and hepatic flow of the Control group recovered only after 24-h leading to the loss of significance between the two groups at this time point (Figure 2) Most prominent between group differences were registered after 6 h of reperfusion (Ileum: RIC_6hours_ vs. Control_6hours_, 132 ± 14 vs. 52 ± 14 %, *p* = 0.001; Liver: RIC_6hours_ vs. Control_6hours_, 102 ± 05 vs. 47 ± 13 %, *p* = 0.001; Figure 2).

Tissue StO_2_ of the liver and ileum showed similar characteristic features, however, StO_2_ values did not follow the positive alterations of flow observed over the course of the reperfusion period in the RIC group (Figure 2). Accordingly, no significant differences were found between the two experimental groups, in terms of StO_2_ (Figure 2).

### 3.2. Biochemical Analysis and Serum Cytokines

**Figure 3:**
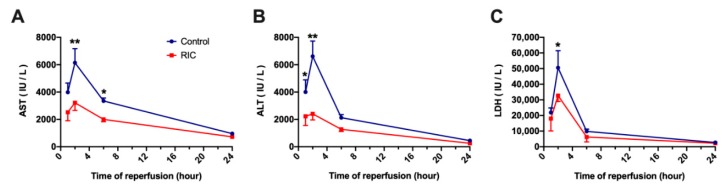
Markers of cellular injury. (**A**–**C**) Time course of transaminases and LDH demonstrated the peak of injury after 2-h of reperfusion. The application of iRIC significantly reduced transaminase and LDH release, especially during the acute phase of reperfusion (mean ± s.e.m., * *p* < 0.05, ** *p* < 0.01, RIC vs. Control, two-way ANOVA and Bonferroni post-hoc test, *n* = 5/group/time point). Abbreviations used: AST—Aspartate aminotransferase; ALT—Alanine aminotransferase; LDH—Lactate dehydrogenase; RIC—Remote ischemic conditioning.

Significant cellular injury has been characterized by markedly increased serum transaminase levels and LDH, showing a peak after 2-h of reperfusion (Figure 3). As it is characteristic following 30-min of THI in rats, both AST and ALT as well as LDH have increased dramatically in the Control group, while the application of iRIC has led to a significantly reduced cellular injury (AST: RIC_2hours_ vs. Control_2hours_, 3217 ± 559 vs. 6145 ± 1025 IU/L, *p* = 0.004; ALT: RIC_2hours_ vs. Control_2hours_, 2409 ± 447 vs. 6613 ± 1117 IU/L, *p* = 0.003; LDH: RIC_2hours_ vs. Control_2hours_, 32,716 ± 1340 vs. 50,578 ± 10 877 IU/L, *p* = 0.03; Figure 3). After a maximal damage following 2-h of reperfusion, a reduction of serum enzyme levels was observed in both groups during the later phase of reperfusion (Figure 3).

**Figure 4:**
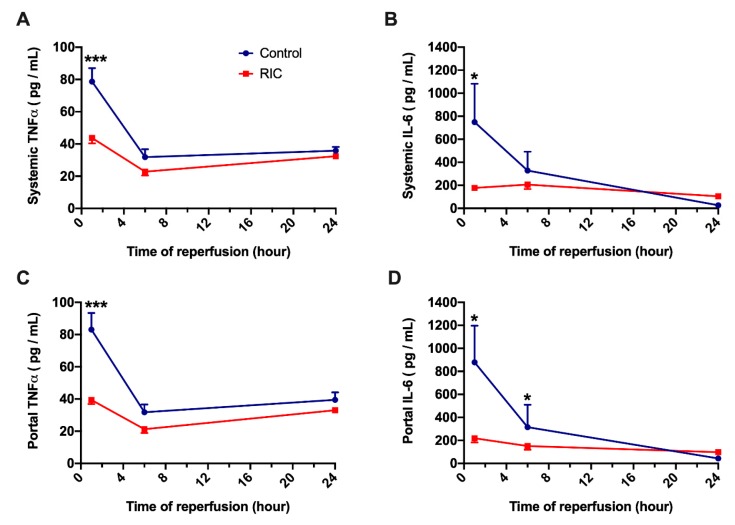
Portal and systemic levels of inflammatory cytokines. No major differences were found between the portal and systemic levels of proinflammatory serum cytokines (TNFα and IL-6). Systemic serum TNFα and IL-6 were significantly elevated in the early phase or reperfusion (1-h) (**A**,**B**). The application of iRIC resulted in a significant decrease of both inflammatory cytokines. Similar changes have been observed in TNFα and IL-6 levels of the portal blood (**C**,**D**) (mean ± s.e.m., * *p* < 0.05, *** *p* < 0.001 RIC vs. Control, two-way ANOVA and Bonferroni post-hoc test, *n* = 5/group/time point). Abbreviations used: IL-6—Interleukin 6; TNFα—Tumor necrosis factor alpha, RIC—Remote ischemic conditioning.

To be able to differentiate between total serum cytokine concentrations and gut related cytokine release, serum levels of TNFα and IL-6 have been measured in both portal- and systemic samples. No major differences were observed between the cytokine levels in these separate samples (Figure 4, A vs. C and B vs. D). During the early phase following THI and liver reperfusion, serum levels of TNFα and IL-6 have increased markedly in the animals of the Control group. In contrast to the Control, iRIC resulted in a reduction of TNFα and IL-6 both in portal and systemic blood, leading to graphically and statistically strongly significant difference between the treated and non-treated groups after 1 h of reperfusion (Systemic TNFα: RIC_1hour_ vs. Control_1hour_, 43.7 ± 3.4 vs. 78.7 ± 8.3 pg/mL, *p* = 0.001; Systemic IL-6: RIC_1hour_ vs. Control_1hour_, 177.6 ± 20.9 vs. 748.7 ± 333.5 pg/mL, *p* = 0.03; Figure 4). However, as it was observed in the characteristics of the serum transaminases, the between group differences have disappeared after 24-h of reperfusion (Figure 4).

### 3.3. Tissue Adenosine Triphosphate Concentration

**Figure 5:**
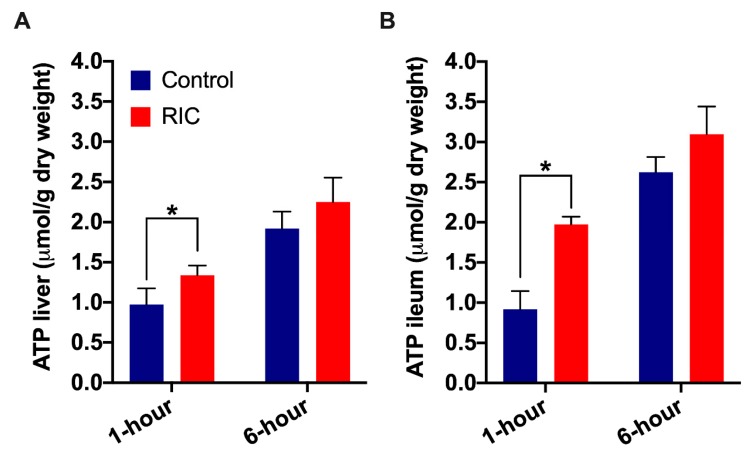
Liver tissue and ileum ATP levels. RIC resulted in better preserved tissue ATP levels throughout the observation period both in the liver (**A**) as well as in the ileum (**B**). Significantly higher hepatic and intestinal ATP levels have been found in the RIC group vs. Control after 1-h of reperfusion. (mean ± s.e.m., * *p* < 0.05 RIC vs. Control, Mann-Whitney-U test, *n* = 5/group/time point). Abbreviations used: RIC—Remote ischemic conditioning; ATP—Adenosine triphosphate.

Similar characteristic features were observed concerning liver and intestinal tissue ATP levels in the RIC and Control groups (Figure 5). After a reduction of ATP levels following 1-h of reperfusion, a substantial recovery of the tissue energy reserves was observed after 3-h. However, the RIC group showed better preserved ATP levels throughout the experiments. There was a significant difference between the RIC and Control groups after 1-h of reperfusion (Liver: RIC_1hour_ vs. Control_1hour_, 1.34 ± 0.12 vs. 0.97 ± 0.20 μmol/g dry weight, *p* = 0.04; Ileum: RIC_1hour_ vs. Control_1hour_, 1.97 ± 0.10 vs. 0.92 ± 0.23 μmol/g dry weight, *p* = 0.02; Figure 5). Despite some graphical differences, no significant disparity was found between the RIC and Control groups after THI and 3-h of reperfusion (Figure 5).

### 3.4. Intestinal Barrier and Bacterial Translocation

**Figure 6:**
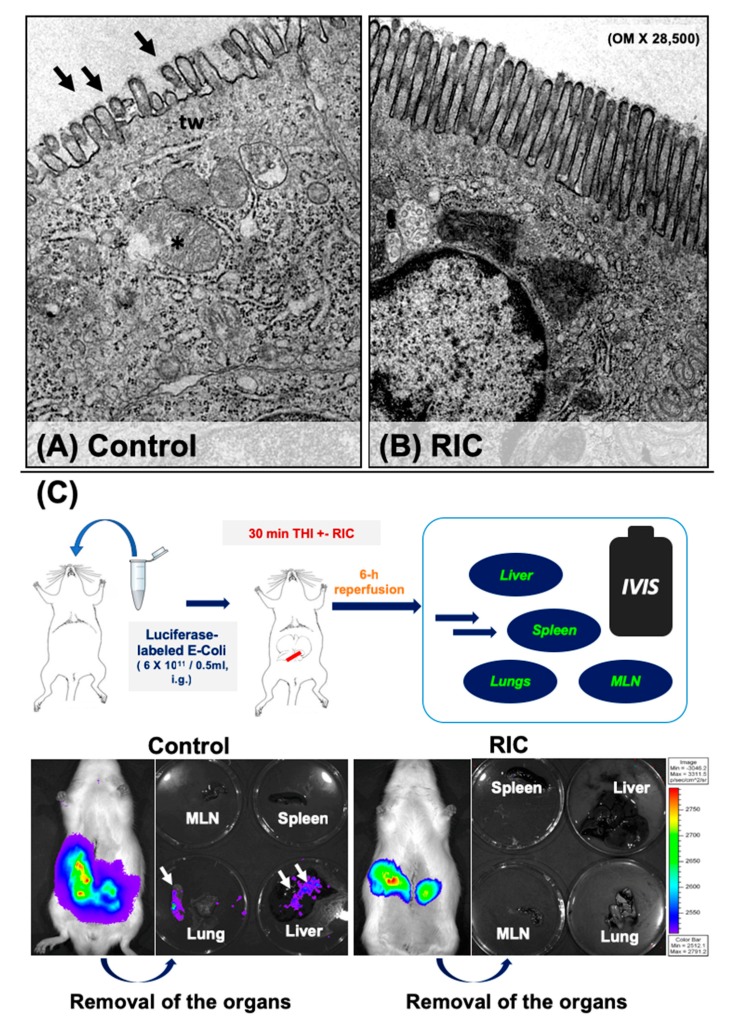
Ultrastructural and functional assessment of intestinal barrier integrity. Following 30-min of total hepatic ischemia and 6-h of reperfusion, electron microscopy of the ileum showed disruption of microvilli which were partially showing signs of vacuolization (arrows) as well as a disintegration of the terminal web (tw) and swollen mitochondria with partial disintegration of their membrane and cristae (asterix) in the animals of the Control group (**A**). Following iRIC, better preserved cellular ultrastructure was observed on the samples of the RIC group with almost regular microvilli and subcellular structures (**B**). To assess functional integrity of the intestinal barrier animals were treated with luciferase labelled *Escherichia coli* before THI (**C**). Following 30-min of ischemia and 6-h of reperfusion, bacterial translocation was assessed using an in vivo imaging system. After the retrieval of the organs these were assessed for luciferase intensity. Intestinal barrier function was better preserved following iRIC and THI. Animals receiving luciferase labelled *E. coli* before IRI had a markedly increased extra-intestinal luciferase activity after 6-h of reperfusion, especially in the liver and the lungs as well as in mesenterial lymph nodes in the Control group. No relevant extra-intestinal activity was observed in the animals of the RIC group. Abbreviations used: OM-original magnification; RIC-remote ischemic conditioning; THI-total hepatic ischemia; IVIS-in vivo imaging system; MLN-mesenterial lymph node.

Ultrastructural analysis of the epithelial layer of the ileum mucosa showed disrupted microvilli which were partially showing signs of vacuolization as well as a disruption of the terminal web and swollen mitochondria with partial disintegration of their cristae and membrane in the animals of the Control group (Figure 6). Following the application of iRIC, much better preserved cellular ultrastructure was observed on the samples of the RIC group with almost regular microvilli and subcellular organelles (Figure 6).

Correlating well with the ultrastructural changes assessed by electron microscopy, intestinal barrier function was better preserved following iRIC and THI. Animals receiving bioluminescent *E. coli* before IRI had a markedly increased extra-intestinal luciferase activity after 6-h of reperfusion, especially in the liver and the lungs as well as in the mesenteric lymph nodes (Figure 6). No relevant extra-intestinal activity was observed in the animals of the RIC group (Figure 6), suggesting the presence of none or only a minor IRI related bacterial translocation.

## 4. Discussion

The present study is one of the first and most comprehensive reports showing a dual protective response triggered by iRIC in a rat model of THI. Our results demonstrate not only the prominent effects of iRIC in mitigating remote hepatocellular damage but also a reduction of local damage of the intestinal barrier induced by severe congestion and functional ischemia in a well-established rodent model of THI. Ultimately, this complex dual protective response triggered by iRIC was manifested in reduced systemic inflammation.

The protective effects of RIC have been reported in IRI scenarios for a diversity of tissues and organs in various experimental and clinical studies over the years [10,11,14,15,16,32,33,34,35]. However, only scarce evidence is available from well-designed and comprehensive experimental studies which would demonstrate the effects of intestinal RIC in total-or partial hepatic ischemia (Table 1). A previous study of our group could show that iRIC can exert potent protection and reduce hepatocellular and intestinal damage following THI via a HO-1 mediated pathway in the second window of protection (using a 48-h recovery period between iRIC and THI) [17]. Here we aimed to assess the acute or so-called “first window” effects of iRIC on hepatocellular injury and intestinal barrier integrity in THI.

As the hepatic vascular bed is located just downstream of the small intestine collecting the portal blood, complete inflow occlusion of the liver without porto-systemic shunt results not only in ischemic liver injury but in severe congestion of the splanchnic organs resulting in functional ischemia of the gut [17]. In this model, the combination of a hepatic IRI and severe intestinal congestion, with structural and functional damage of the intestinal barrier and consequential bacterial translocation, are leading to a systemic proinflammatory activation [17,36]. We hypothesize that the benefit of iRIC in the scenario of THI, compared to the more widely used and reported RIC of the limbs, lies in the combination of a “local conditioning” on the intestine which may protect against the detrimental intestinal congestion and a “remote conditioning” effect targeting the liver.

Impairment of tissue microcirculation is one of the key elements in IRI [10,11,37]. A combination of different mechanisms is contributing to a post-ischemic microcirculatory failure such as endothelial neutrophil stasis, cell swelling, sludges and formation of micro-thromboses in small capillaries and liver sinusoids [11,37]. In the present study we registered hepatic and intestinal microcirculation using the laser Doppler based O2C system. Remote conditioning resulted in better preserved microcirculation of the liver and the gut, especially during the early phase of liver reperfusion, however, the significant difference on the level of microcirculation disappeared after 24-h of reperfusion. Microcirculatory failure is not only a consequence of IRI but it also actively contributes to the paradox damage documented as reperfusion injury by maintaining an impaired perfusion at the level of the small capillaries and sinusoids and aggravating tissue injury [37]. Positive effects of RIC on target organ circulation were confirmed in various IRI models [11,13,14,15,38,39]. In previous reports, we could demonstrate that RIC applied on the infrarenal aorta is able to potently improve graft macro- and micro-circulation including post-reperfusion microcirculatory flow and portal venous flow in a rat model of orthotopic liver transplantation as well as in 70% partial liver ischemia and liver resection [11,13,14,15].

A sublethal period of 30-min THI in rats without porto-systemic shunt leads to a severe hepatocellular damage, characterized by a prominent elevation of serum transaminases [40]. In our study the peak of hepatocellular injury was observed 2-h following liver reperfusion with strongly increased AST, ALT and LDH levels. The potent ability of iRIC to mitigate hepatocellular injury was characterized by significantly reduced transaminases and LDH in the RIC group compared to the non-treated animals. These findings are in line with our previous reports with limb RIC and partial liver ischemia [13,14] or liver transplantation [11,15] as well as correlates with our findings with iRIC and THI showing similar effects in the second window of protection after 48-h [17].

A rapid drop of tissue ATP content during THI results in disturbed active ion transport mechanisms, contributing to cellular swelling, microcirculatory failure and cell death [11,37,41]. A “reconditioning” effect, leading to better preserved or increased ATP production is associated with improved mitochondrial and cellular integrity following RIC and ischemia (via the prevention of mitochondrial permeability transition pore opening, less mitochondrial oxidative stress, reduction of calcium overload) [42,43]. Therefore, increased levels of tissue ATP may be interpreted as a global manifestation of better preserved mitochondrial and cellular functions. Previous reports could show the beneficial effects of different ischemic conditioning approaches and pharmacological agents on tissue energetic status and ATP levels [11,44,45]. Our data show favorable alterations in hepatic and intestinal tissue ATP levels during reperfusion and iRIC. After 1-h of reperfusion significantly higher ATP levels were found in the RIC group vs. Control.

There is a plethora of experimental and clinical evidence showing that imbalance in systemic pro- and anti-inflammatory processes likewise belongs to the main events in the pathophysiology of liver IRI [11,46]. An inflammatory cytokine release, associated with intestinal congestion and ischemia as well as bacterial translocation further aggravate systemic and remote organ damage leading to inferior outcomes [47,48]. In previous reports we could show that RIC of the limbs results in the upregulation and downregulation of anti- and pro-inflammatory cytokines (including Interleukin-10, Monocyte chemoattractant protein-1, Macrophage Migration Inhibitory factor, and TNFα) in models of orthotopic liver transplantation or partial liver ischemia [11,14,15]. In line with these previous findings, our present results show significantly reduced levels of TNFα and IL-6 in portal- and systemic blood, suggesting a greatly reduced systemic inflammation following the application of iRIC in this severe model of THI. The comparable levels of both cytokines observed in the portal- and systemic circulation indicates that the gut is the major source of these cytokines [36].

However, a significant elevation in the serum levels of inflammatory cytokines is not only a sign of increased tissue damage and systemic inflammation, but these cytokines also actively participate in aggravating local tissue injury induced by THI [36]. The increase in translocation of bacterial products to the gut-associated lymphatic tissue following hypoxia/ischemia of the intestinal wall tissue triggers an orchestra of pro-/anti-inflammatory cytokine release [36,49,50]. Among these inflammatory mediators, TNFα has received an intense scientific interest because of its role in increasing tight-junction (TJ)-permeability in the intestine not only through a decreased expression of TJ proteins but via the activation of myosin light chain kinases, leading to a disruption of barrier function [36,49]. Therefore, enhanced TNFα levels appears to play a central role in promoting pathological bacterial translocation [36].

Other cytokines such as IL-6 and Interferon-gamma have been shown to increase intestinal epithelial permeability and induce translocation of *E. coli* across epithelial cells [36,51]. Accordingly, we have observed a relevant ultrastructural damage of the epithelial cells of the ileum following 30-min of THI and 6-h of reperfusion in our present model, including a disruption of the microvilli, disintegration of the terminal web structure and swollen mitochondria with partial disintegration. Following iRIC these pathological alterations were largely mitigated with an overall better preserved cellular ultrastructure.

These structural observation correlated well with the observed damage of the functional barrier. Following the administration of bioluminescence *E. coli*, significant extra-intestinal accumulation was observed in the animals of the Control group, while no relevant luciferase activity was detected by the IVIS system in the animals of the RIC group, suggesting an active reduction of bacterial translocation by iRIC. Only very limited data is available to date on the effects of iRIC on barrier integrity and bacterial translocation following liver IRI (Table 1). In our previous report by Kageyama et al., we could show a reduced mucosal damage in the second window of protection following iRIC assessed by the Park score on conventional histological samples [17]. This was associated with an increased expression of hemoxygenase-1 (HO-1) in the intestinal mucosa and in the liver, suggesting a potential mechanistic role of HO-1 behind the effects of late iRIC [17].

As the presents study did not aim to compare different iRIC protocols concerning the length and numbers of cycles, it is not possible to draw a conclusion, whether there are alternative iRIC protocols potentially triggering an even superior protective response. Despite the many reports attempting to find the optimal RIC protocol, a widely accepted guideline is still not within reach and the selection of the protocols is mostly based on empiric choices [10,52,53]. Due to the lack of an optimal RIC protocol some authors have also expressed their concern about a potential “hyperconditioning” phenomenon, where excessive or repetitive stimulus may lead to a deleterious effect [52,54]. While the skeletal muscle of the extremities (where RIC is conventionally applied) has a high ischemic tolerance, the small bowel mucosa is notoriously sensitive for hypoxia and ischemia [10,11,14,15]. Therefore, in the present study we aimed to use a less intense protocol with shorter total ischemia times (8 min in total) to minimize the ischemic insult of the small bowel but maximize the potential benefits.

The findings of our study have to be interpreted in the light of certain limitations. This study is one of the first reports on iRIC in THI (Table 1). Therefore, in this preliminary setting we were not able to perform a deep exploration of the subcellular mechanisms behind the observed protective effects of iRIC, thus our findings remain partially descriptive. The further inclusion of an “iRIC only” (an experimental group receiving iRIC treatment without THI) and sham laparotomy groups may have strengthened the statistical analysis and the conclusions of the study, resulting in a more solid overall message.

**Figure 7:**
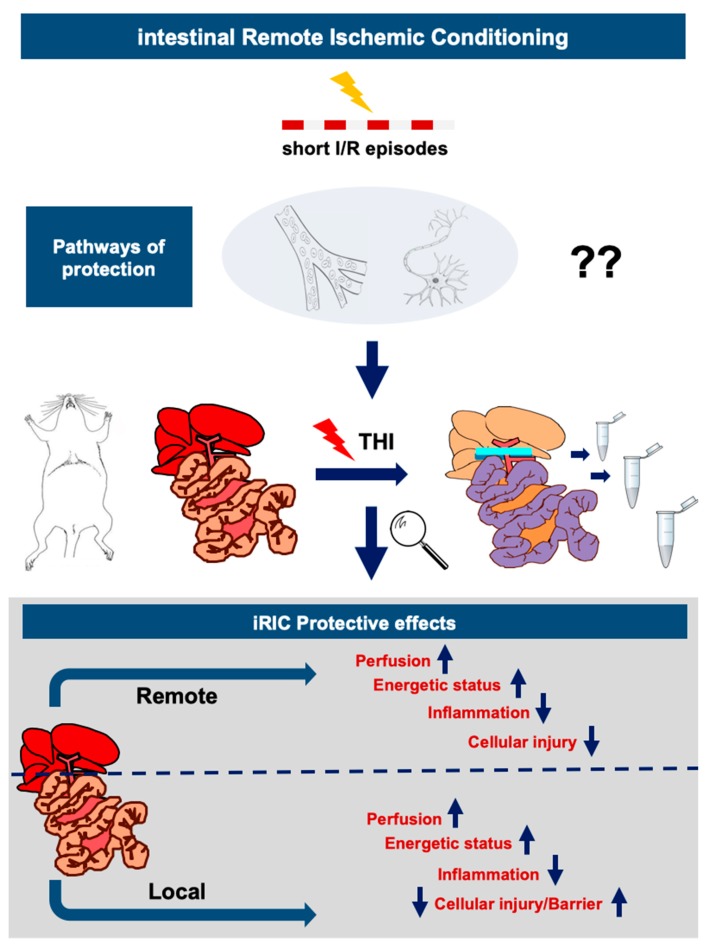
Summary of the mechanism and effects of iRIC observed in the setting of THI in rats. The following flowchart depicts the observed and possible protective effects and mechanisms of action of iRIC following THI in rats. Briefly, iRIC applied as short periods of ischemia-reperfusion before THI at a remote organ (intestine) results in the transfer of protective signals via different humoral and/or neural and partially unknown connective mechanisms to the target organ (liver), however, it seemingly also confers a local protection against the detrimental effects of intestinal congestion and functional ischemia of the gut, via preserving the integrity of the intestinal tissue and barrier function and resulting in less dramatic systemic effects of THl. Adapted from Emotzpohl, Czigany et al. Shock. 2018 [15]. Abbreviations used: iRIC—Intestinal remote ischemic conditioning; THI—Total hepatic ischemia.

Notwithstanding the aforementioned limitations, the present study shows some novel findings on the dual way of protection conferred by iRIC in a well-established model of THI without porto-systemic shunt. iRIC seemed to be a feasible technique which could potently reduce hepatocellular injury, improve intestinal and hepatic microcirculation, positively influenced inflammatory cytokine and tissue ATP levels and mitigated a consequential disintegration of the intestinal barrier and prevented bacterial translocation (Figure 7). More detailed exploration of the mechanistic steps behind these observations and identification of the connecting pathways between the gut and the liver following iRIC and THI would be of interest for future basic and translational research.

## Figures and Tables

**Table 1:** Studies with intestinal remote ischemic conditioning in liver ischemia.

Author	Species and Strain	Model	Sample Size	RIC Protocol	Time Points	Short Summary
*Czigany* et al. *(present study)*	Rat, Wistar, outbred, male	30 min THI	50 rats, 25 RIC	Remote preconditioning, 2 × 4 min of ischemia and 11 min of reperfusion by clamping the SMA directly before THI	1, 2, 6, 24 h	iRIC is a feasible technique which could potently reduce hepatocellular injury and preserve intestinal barrier integrity following THI
*Kageyama* et al. [17]	Rat, Wistar, outbred, male	30 min THI	36 rats, 18 RIC	Second window remote preconditioning, 2 × 4 min of ischemia and 11 min of reperfusion by clamping the SMA 48 h before THI	2, 6, 24, 240 h	iRIC remarkably attenuates hepatic IRI in the second window of protection after 48-h, presumably by HO-1 induction in hepatocytes
*Xue* et al. [18]	Rat, Sprague-Dawley, outbred, male	30 min, 70% partial ischemia	15 rats, 5 RIC	Remote preconditioning, 2 × 10 min of ischemia and 10 min of reperfusion by clamping the SMA directly before THI	3 h	iRIC provided protection against hepatic IRI by modulating apoptosis and inflammation.

Literature search (PubMed; search date: 10 September 2019; search terms: ischemic preconditioning OR ischemic conditioning AND intestine AND liver) resulted in two relevant studies on iRIC in liver ischemia, showing very limited evidence available on the effects of iRIC in THI. According to the best of our knowledge, the present study is one of the most comprehensive experimental works so far on iRIC and its effects in liver IRI. Abbreviations used: THI—Total hepatic ischemia; RIC—Remote ischemic conditioning, IRI—Ischemia reperfusion injury; SMA—Superior mesenteric artery.
